# Association Between Serum Ionized Calcium Levels and Neurological Outcomes in Patients with Out-of-Hospital Cardiac Arrest

**DOI:** 10.3390/life15121889

**Published:** 2025-12-10

**Authors:** Shin Young Park, Hyun-Soo Zhang, Incheol Park, Je Sung You, Yoo Seok Park

**Affiliations:** 1 Department of Emergency Medicine, Yonsei University College of Medicine, 50 Yonsei-ro, Seodaemun-gu, Seoul 03722, Republic of Korea; chilli89@yuhs.ac (S.Y.P.); incheol@yuhs.ac (I.P.); youjsmd@yuhs.ac (J.S.Y.); 2 Biostatistics Collaboration Unit, Department of Biomedical Systems Informatics, Yonsei University College of Medicine, 50 Yonsei-ro, Seodaemun-gu, Seoul 03722, Republic of Korea; hszhang@yuhs.ac

**Keywords:** calcium, ionized, out-of-hospital cardiac arrest, neurological outcomes

## Abstract

Despite advances in post-cardiac arrest care, mortality and poor neurological outcomes remain common after out-of-hospital cardiac arrest (OHCA). Calcium imbalance is characteristic of post-cardiac arrest syndrome, but its prognostic role is unclear. We retrospectively analyzed 421 OHCA patients treated with targeted temperature management (TTM) (2011–2023). pH-adjusted ionized calcium levels were measured at 0, 12, 24, 48, and 72 h after return of spontaneous circulation (ROSC). Associations with 30-day neurological outcomes and mortality were assessed using multivariable logistic regression with two-stage maximum likelihood estimation. Higher baseline-adjusted ionized calcium levels were significantly associated with better neurological outcomes (Cerebral Performance Category 1–2) and lower 30-day mortality, regardless of calcium infusion or clinical covariates. Each 0.01-unit increase corresponded to 17% lower odds of unfavorable neurological outcome (odds ratio [OR], 0.83; 95% confidence interval [CI], 0.76–0.90) and 10% lower mortality (OR, 0.90; 95% CI, 0.84–0.96). Incorporating longitudinal calcium measurements improved predictive accuracy, raising the area under the curve for neurological outcomes from 0.843 to 0.919. Early post-ROSC ionized calcium levels were independently associated with neurological outcomes and mortality in patients with OHCA treated with TTM. Serial ionized calcium monitoring may serve as a prognostic marker, warranting prospective evaluation of therapeutic implications.

## 1. Introduction

Despite advances in post-cardiac arrest care, in-hospital mortality remains high among patients who experience out-of-hospital cardiac arrest (OHCA) [1,2,3]. Post-cardiac arrest syndrome involves a complex interplay of pathophysiological processes following the return of spontaneous circulation (ROSC), including post-cardiac arrest brain injury, myocardial dysfunction, systemic ischemia–reperfusion injury, and effects of the initial cause of arrest [4]. Among these, systemic ischemia–reperfusion injury contributes substantially to multi-organ dysfunction and plays a critical role in neurological injury after ROSC [5].

Under normal physiological conditions, ionic homeostasis is maintained by adenosine triphosphate (ATP)-dependent membrane transporters. Ischemia leads to anaerobic metabolism, lactate accumulation, and ATP depletion [6], which impair ion pumps and cause abnormal ion shifts between intra- and extracellular spaces [7,8]. During reperfusion, restored blood flow generates reactive oxygen species, which further disturb calcium homeostasis by increasing intracellular ionized calcium levels, ultimately resulting in mitochondrial dysfunction, membrane failure, and cell death [9,10].

The disruption of calcium homeostasis has been implicated in the pathogenesis of cerebral ischemia [11,12]. Animal studies have demonstrated that oxidative stress, inflammation, and calcium-binding proteins are involved in calcium-mediated neuronal injury [13]. An early study in feline models reported that failure to restore brain electrical activity after prolonged ischemia was associated with sustained elevations in brain tissue calcium levels, suggesting a link between calcium accumulation and irreversible neuronal damage [14]. Clinical studies have also emphasized the relevance of calcium imbalance; a multicenter study using the eICU Collaborative Research Database found that abnormal serum calcium levels, particularly hypocalcemia, were associated with an increased risk of cardiac arrest in patients with acute stroke [15]. Similarly, a retrospective analysis from the Medical Information Mart for Intensive Care IV database reported that reduced serum calcium levels were independently associated with poor outcomes in critically ill patients with acute ischemic stroke [16]. Collectively, these findings highlight the potential clinical and prognostic significance of calcium dysregulation in ischemic brain injuries. However, only a limited number of studies have specifically evaluated the association between ionized calcium levels and outcomes in patients with post-ROSC OHCA. Kim et al. [17] reported that higher ionized calcium levels at emergency department arrival were associated with an increased likelihood of achieving ROSC but not with survival or neurological outcomes. In contrast, Akasaka et al. [18] found that the lowest pH-adjusted ionized calcium concentration within 48 h post-ROSC was significantly lower in patients with unfavorable neurological outcomes. These divergent results underscore the need for further investigation of the temporal dynamics and prognostic implications of ionized calcium in this population. Therefore, this study aimed to evaluate the relationship between adjusted ionized calcium levels and neurological outcomes in patients treated with targeted temperature management (TTM) following ROSC after OHCA.

## 2. Materials and Methods

### 2.1. Study Design and Population

We conducted a retrospective analysis using prospectively collected data from the registry of a critical pathway (CP) designed for post-resuscitation care, including TTM. These data were sourced from Yonsei University College of Medicine, Severance Hospital, a tertiary hospital located in Seoul, Korea, from September 2011 to September 2023. Patients who successfully regained spontaneous circulation after OHCA were eligible for activation of the CP, based on predefined inclusion and exclusion criteria [19]. We gathered data on the index cardiac arrest event and its outcomes following the Utstein Style recommendation for reporting cardiac arrest research [20]. Post-cardiac arrest care, including TTM, was administered according to established international guidelines. Patients aged <19 years, those who died within 24 h of ROSC, those whose families asked for withdrawal of life-sustaining treatment during TTM, and those who were administered intravenous calcium infusion during cardiopulmonary resuscitation (CPR) were excluded from the analysis.

Patients were provided post-cardiac arrest care based on recent guidelines [21,22]. All patients who were unable to follow commands were treated with TTM with a target core body temperature between 33 °C and 36 °C, with the exception of those with active bleeding, refractory hemodynamic instability, and possible noncardiac causes of coma or terminal malignancy.

The study was reviewed and approved by the Institutional Review Board of Yonsei University College of Medicine, Severance Hospital (approval reference no. 4-2024-1000). Owing to the retrospective design of the study, the ethics committee waived the requirement for informed consent.

### 2.2. Data Collection

We collected the following data: patients’ demographic data (age, sex, and comorbidities) and resuscitation variables (initial monitored rhythm, presence of a witness on collapse, bystander CPR, time from collapse to ROSC, presence of defibrillation, total dose of epinephrine during CPR, and cause of arrest). The use of vasoactive agents, extracorporeal membrane oxygenation, and continuous renal replacement therapy was also recorded. Post-cardiac arrest shock was defined as the requirement for vasopressors or inotropes to sustain a mean arterial pressure of >65 mmHg despite fluid administration within 72 h of cardiac arrest.

### 2.3. Assessment of Serum Ionized Calcium

Based on the CP protocol of our institution, ionized calcium levels were measured immediately, 12 h, 24 h, 48 h, and 72 h after ROSC. We also collected the levels of serum electrolytes (sodium, potassium, chloride, phosphate, calcium, and magnesium) and the parameters that might affect electrolyte levels (pH and albumin). The pH-adjusted ionized calcium concentration is widely regarded as the most accurate indicator of physiologically active calcium. Accordingly, trends in adjusted ionized calcium were analyzed using blood gas data as follows [18]:

Adjusted ionized calcium = actual ionized calcium × (1 − 0.53 [7.4 − actual blood pH])

This adjustment was applied because arterial pH has a strong influence on measured ionized calcium levels: acidemia increases measured ionized calcium by reducing protein binding, whereas alkalemia decreases it. The administration of calcium is not included in the established international guidelines or CP protocol of our institution; therefore, the decision was made at the discretion of the attending physician.

### 2.4. Outcome Measures

The primary outcome of this study was the neurological outcome at 30 days after ROSC. The Cerebral Performance Category (CPC) score was used to assess neurological outcome, with a CPC score of 1–2 considered indicative of a favorable neurological outcome, whereas a score of 3–5 was deemed reflective of an unfavorable neurological outcome [23]. The secondary outcome was the 30-day mortality. In accordance with previous cardiac arrest studies, death was scored as 5 on the CPC scale; therefore, neurological outcomes were evaluated for all 421 patients, including those who died within 30 days after ROSC.

### 2.5. Statistical Analysis

Baseline characteristics of the study population were summarized using mean ± standard deviation or median (interquartile range) for continuous variables and frequency (%) for categorical variables. Comparisons by outcome status were performed using the chi-square test or Fisher’s exact test for categorical variables and the independent *t*-test or Wilcoxon rank-sum test for continuous variables, as appropriate.

As both the primary and secondary outcomes were binary, a logistic regression analysis was conducted. Variables with a *p*-value <0.10 in univariable analyses were considered candidates for multivariable modelling. Further variable selection employed Akaike information criterion (AIC)-based stepwise selection and least absolute shrinkage and selection operator methods [24,25]. The adjusted ionized calcium measure, which is the main predictor of interest, was included in the multivariable models by default. Clinical and epidemiological judgments were also applied to finalize the multivariable models.

Given the repeated measures of adjusted ionized calcium over time (0, 12, 24, 48, and 72 h post-ROSC), two separate analyses were conducted. First, each time period’s adjusted ionized calcium measurement was individually entered into separate multivariable logistic regression models, adjusting for the corresponding prior period’s calcium infusion dose. The models were further adjusted for other potential risk factors (identified from the variable selection outlined above), including initial rhythm, time-to-ROSC, non-cardiac cause, renal event, sex, and witnessed status for the primary outcome (CPC), and initial rhythm, time-to-ROSC, non-cardiac cause, renal event, and witnessed status for the secondary outcome (mortality). Model goodness-of-fit was evaluated using the AIC, and predictive accuracy was compared using the area under the receiver operating characteristic curve (AUC). Differences in AUC were assessed using DeLong’s test [26]. Second, to simultaneously account for all repeated ionized calcium measurements, a two-stage maximum likelihood estimation (MLE) approach was implemented [27,28,29]. Initially, a linear mixed model (LMM) with random intercepts and slopes over time was applied to the longitudinal ionized calcium data for each patient, with the prior longitudinal period’s calcium infusion dose as a predictive factor of longitudinal ionized calcium levels [30]. Subsequently, the estimated random intercept (baseline ionized calcium levels at 0 h post-ROSC) and random slope (rate of change in ionized calcium levels over time) were entered into multivariable logistic regression models, adjusting for total (cumulative) calcium infusion dose within the first 72 h post-ROSC, and other potential risk factors according to each outcome (CPC, mortality).

All statistical tests were two-sided, and statistical significance was defined as a *p* < 0.05. Analyses were conducted using SAS version 9.4 (SAS Inc., Cary, NC, USA) and R version 4.4.3 (R Foundation for Statistical Computing, Vienna, Austria).

## 3. Results

### 3.1. Baseline Characteristics of the Study Population

**Figure 1:**
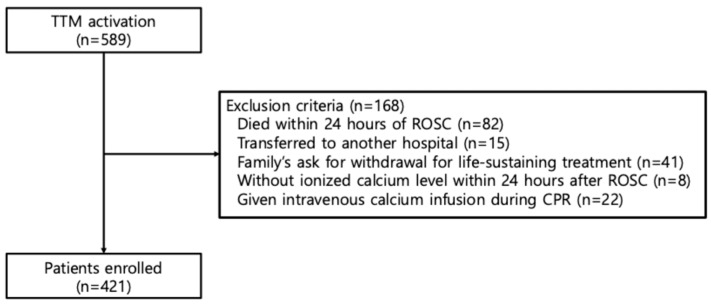
Study flow diagram. TTM, targeted temperature management; ROSC, return of spontaneous circulation; CPR, cardiopulmonary resuscitation.

Between September 2011 and September 2023, 589 patients who achieved ROSC after OHCA underwent TTM. After excluding 168 patients based on predefined criteria, 421 patients were included in the final analysis (Figure 1). The baseline characteristics and laboratory data of the patients are summarized in Table 1.

Regarding the primary and secondary outcomes, 240 (57.1%) patients had an unfavorable neurological outcome (CPC of 3–5) at 30 days post-ROSC, and 158 (37.5%) patients died within 30 days. Demographic variables, cardiac arrest information, comorbidities, intensive care information, adjusted ionized calcium measurements over time, calcium infusion Y/N, and total calcium infusion dose that differed significantly in univariable analyses are shown in Table 1.

### 3.2. Comparison of Model Performance (AIC and AUC) Among Models, Including Adjusted Ionized Calcium Measurements at Different Time Points (0, 12, 24, 48, and 72 h Post-ROSC), Controlling for the Corresponding Prior Time Period’s Calcium Infusion Dose

Table 2 summarizes the comparative performance of six multivariable logistic regression models for each outcome, differing by their inclusion of adjusted ionized calcium measurements at different time points (0, 12, 24, 48, and 72 h post-ROSC) or without any calcium-related variables. Model performance was assessed using AIC goodness-of-fit and AUC predictive accuracy.

Including adjusted Ca^2+^ while controlling for the prior calcium infusion dose at any time point significantly improved AIC and AUC compared to those with the model without calcium-related variables for both neurological and mortality outcomes at 30 days. Among the models including calcium-related variables, adjusted Ca^2+^ levels measured at 48 h post-ROSC showed the best AIC and AUC for the neurological outcome, whereas adjusted Ca^2+^ measured at 12 h post-ROSC provided the best performance for the mortality outcome. Detailed results for all 12 multivariable logistic regression models (six for each outcome) are available as Supplementary Material (Tables S1 and S2).

### 3.3. Final Multivariable Models Incorporating Longitudinal Adjusted Ionized Calcium Levels, While Controlling for Total Calcium Infusion Dose

Two-stage MLE modelling enabled the incorporation of all available longitudinal information on adjusted ionized calcium measurements and calcium infusion doses. Each participant’s estimated random intercept (baseline ionized calcium levels at 0 h post-ROSC) and random slope (rate of ionized calcium change over time) from the first-stage LMM were included in subsequent multivariable logistic regression models. Additionally, the total (cumulative) calcium infusion dose within the first 72 h post-ROSC and other potential risk factors identified through variable selection were adjusted for.

The results of the final multivariable models are presented in Table 3, along with the univariable model results for each variable included in the multivariable model. The variable ‘sex’ was excluded from the multivariable model for 30-day mortality following variable selection. For the neurological (CPC) outcome at 30 days, baseline-adjusted ionized calcium levels were significantly associated with the outcome (*p* < 0.001). Each 0.01-unit increase in baseline-adjusted ionized calcium levels corresponded to 17% lower odds (odds ratio [OR], 0.83; 95% confidence interval [CI], 0.76–0.90) of an unfavorable neurological outcome, while controlling for total calcium infusion dose. The rate of change in adjusted ionized calcium levels was not significantly associated with the outcome (OR, 1.04; 95% CI, 0.78–1.38) after adjusting for other variables. Similar findings were observed for 30-day mortality, although the effect sizes were only slightly attenuated. Baseline adjusted ionized calcium levels (OR, 0.90; 95% CI, 0.84–0.96; *p* = 0.001) were a significant predictor of lower mortality odds, adjusting for total calcium infusion dose and other risk factors. Again, the rate of change in adjusted ionized calcium levels over time was not significantly associated with mortality (OR, 1.02; 95% CI, 0.82–1.27) after adjustment.

### 3.4. Trends of Mean Adjusted Ionized Calcium Levels over Time by Outcome Status

**Figure 2:**
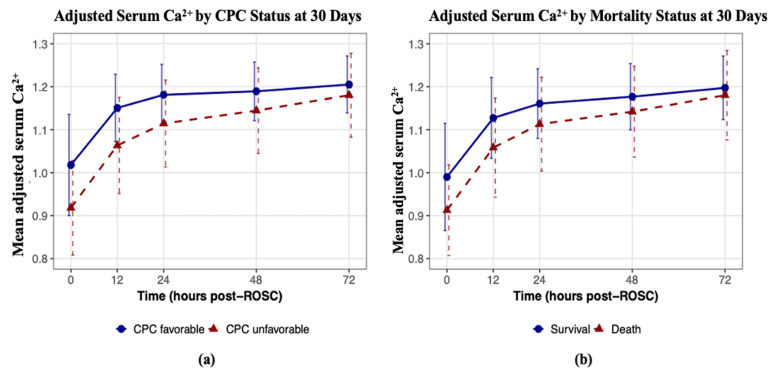
Mean trajectories of adjusted ionized calcium levels over 72 h post-ROSC, stratified by outcome. (**a**) Patients with favorable neurological outcomes at 30 days maintained higher mean adjusted ionized calcium levels than did those with unfavorable outcomes, despite similar increasing trends. (**b**) Survivors at 30 days post-ROSC also presented consistently higher calcium levels than those of non-survivors, with both groups exhibiting parallel increases over time. ROSC, return of spontaneous circulation; CPC, Cerebral Performance Category.

Figure 2 depicts the mean trajectories of adjusted ionized calcium levels over 72 h post-ROSC by each outcome status (CPC, mortality). Patients with favorable neurological outcome at 30 days (CPC 1–2) and those who survived to 30 days post-ROSC consistently maintained higher mean adjusted ionized calcium levels over time, although the upward trend in adjusted ionized calcium levels was similar between the outcome groups.

The graphical trends confirm findings from the final multivariable models in Table 3, with the greatest difference in mean adjusted ionized calcium levels between favorable and unfavorable outcome groups observed at baseline (0 h post-ROSC), with differences diminishing over time owing to a slightly steeper increase in the unfavorable outcome group. This observation aligns with the multivariable OR estimates and corresponding *p*-values in Table 3, where higher baseline adjusted ionized calcium levels were strongly associated with favorable outcomes (ORs < 1), whereas the rate of adjusted ionized calcium change showed no significant associations (ORs slightly > 1).

### 3.5. Comparison of AUC Among Multivariable Models with Different Levels of Information Regarding Longitudinal Adjusted Ionized Calcium Measurements

Table 4 summarizes the results of DeLong’s test on AUC (predictive accuracy) differences among different logistic regression models for each outcome. The compared models were model 1: without calcium-related variables, model 2: single most predictive time point’s adjusted ionized calcium levels, controlling for corresponding prior calcium infusion dose, and model 3: full longitudinal information of adjusted ionized calcium levels, controlling for the total calcium infusion dose.

For the neurological (CPC) outcome at 30 days, AUC improved significantly as more information regarding longitudinal adjusted ionized calcium measurements was included. AUC increased from 0.84 (95% CI, 0.80–0.88) without calcium-related variables to 0.88 (95% CI, 0.85–0.92) with data from a single time point and further to 0.92 (95% CI, 0.89–0.94) with complete longitudinal data (all DeLong’s test *p* ≤ 0.005).

For the 30-day mortality outcome, including calcium-related variables significantly improved compared to that with no inclusion (*p* = 0.012). However, further inclusion of all longitudinal calcium-related data provided a modest, non-significant improvement compared with that including calcium-related data from a single time point (*p* = 0.124).

## 4. Discussion

In this retrospective cohort study of patients with OHCA treated with TTM, higher adjusted ionized calcium levels during the first 72 h post-ROSC were significantly associated with favorable neurological outcomes and lower 30-day mortality. These associations remained robust after adjusting for calcium infusion and clinical covariates, suggesting an independent prognostic role for calcium homeostasis in post-cardiac arrest care. Given the observational design of this study, the relationship between ionized calcium levels and outcomes should be interpreted as an association rather than causation. Moreover, current resuscitation guidelines do not recommend routine calcium administration in post-cardiac arrest care; therefore, our findings should be considered hypothesis-generating rather than practice-changing.

This study provided three key insights. First, although the association between adjusted calcium levels and outcomes was consistently observed at all measured time points (0, 12, 24, 48, and 72 h post-ROSC), the magnitude of predictive performance differed depending on when the measurement was taken. This finding suggests that, although calcium levels maintain prognostic value over time, the strength of their association with outcomes may vary slightly across different phases of post-resuscitation care. Second, our two-stage modelling showed that baseline-adjusted calcium was a strong and consistent predictor of both neurological outcomes and mortality, whereas the rate of calcium change (slope) was not significantly associated with outcomes. The absence of a prognostic association for the calcium slope may reflect the influence of exogenous factors—such as calcium infusion, fluid therapy, and acid–base fluctuations—which can cause short-term variability in ionized calcium levels and obscure intrinsic cellular recovery processes. This does not undermine the value of the serial measurements, which are necessary to estimate the true baseline levels under dynamic conditions following ROSC. Even after accounting for calcium infusion in our models, the slope did not show a significant prognostic value, which may indicate that dynamic calcium trends are less reflective of intrinsic recovery than are static baseline levels. Finally, incorporating longitudinal calcium data significantly improved the model performance. For neurological outcomes, the AUC increased from 0.84 (no calcium data) to 0.88 (single time point) to 0.92 (full serial data) as more data were added to the model. A similar, albeit smaller, improvement in mortality was observed. These findings underscore the importance of serial ionized calcium monitoring in the early post-ROSC period, as longitudinal data provide the greatest prognostic accuracy, particularly for neurological outcomes.

The biological plausibility of our findings is supported by known mechanisms of calcium dysregulation in post-cardiac arrest syndrome. Global ischemia followed by reperfusion leads to cellular energy failure, impaired ATPase-dependent ion transport, and intracellular calcium overload, which in turn trigger mitochondrial dysfunction, oxidative stress, activation of proteolytic enzymes, and ultimately, neuronal apoptosis or necrosis [6,31]. Neurons are also particularly vulnerable to calcium-mediated excitotoxicity during ischemic injury [32]. These pathophysiological processes form the mechanistic basis of the observed associations between calcium levels and outcomes. This has led to continuous research on the role of calcium in neural signaling, particularly its relationship with neurological outcomes in various diseases [33,34,35,36].

Previous studies examining calcium and post-arrest prognosis have largely focused on total serum calcium [37,38], which is affected by albumin level, acid–base status, and other confounders [39]. In contrast, ionized calcium reflects the biologically active fraction and directly affects cardiac contractility, vascular tone, and neural excitability [40,41,42,43,44,45]. Calcium formulations commonly administered intravenously in clinical settings, such as calcium chloride or calcium gluconate, also provide ionized calcium, the physiologically active form that acts immediately without requiring metabolic conversion [46]. Therefore, in this study, we used ionized calcium level as the principal measure of calcium status. As ionized calcium levels are strongly pH-dependent [47], we applied pH-adjusted ionized calcium values standardized to 7.4 to improve measurement accuracy and minimize confounding [48].

Current CPR and post-resuscitation guidelines do not recommend routine calcium administration owing to a lack of evidence for clinical benefit [49,50,51,52]. These recommendations are largely based on small studies or case series that focus on the total calcium concentrations. The distinction between total and ionized calcium is crucial, as only the latter exerts physiological effects and may better reflect the severity of critical illness. In our study, calcium administration was not guided by a standardized protocol but was left to the discretion of the treating physician. To mitigate this potential source of bias, we adjusted for both time point-specific and cumulative calcium infusion doses in our multivariable analyses. Although calcium infusion may have interacted with outcome trajectories, our findings indicate that the association between higher adjusted ionized calcium levels and favorable outcomes remained independent of supplementation, even after adjusting for infusion dose. Nevertheless, residual confounding cannot be fully excluded. To further assess whether calcium supplementation was selectively administered to more severely ill patients, we compared total calcium infusion doses across two surrogate severity indicators: cardiac failure and continuous renal replacement therapy. The mean infusion dose did not differ significantly according to either indicator (*p* = 0.064 and *p* = 0.427, respectively ). Furthermore, incorporating these variables into the multivariable model did not alter the association between baseline ionized calcium and outcomes.

To address the possibility that other electrolytes might confound the association between ionized calcium and clinical outcomes, we examined whether additional biochemical variables could influence this relationship. Although these electrolytes are physiologically linked to calcium homeostasis, a Pearson correlation analysis demonstrated substantial inter-correlation among several of them—particularly among divalent cations and acid–base-related parameters—raising concerns regarding multicollinearity. For this reason, these variables were not included in the primary multivariable models, which were intentionally designed to preserve a clear calcium-focused analytic framework. Nevertheless, we evaluated their potential influence using extended regression analyses that incorporated all seven laboratory parameters, including electrolytes (sodium, potassium, chloride, magnesium, phosphate) as well as albumin and lactate. Importantly, the association between baseline ionized calcium and clinical outcomes remained essentially unchanged, indicating that the primary findings were not driven by confounding from these additional physiological factors (Tables S3 and S4). Given the following—(a) the substantial inter-correlation among multiple electrolytes and the resulting risk of multicollinearity if included in the primary models; (b) the conceptual need to maintain a calcium-specific modeling strategy; and (c) the consistent robustness of baseline ionized calcium in sensitivity analyses—we believe that a simpler and more parsimonious calcium-centered model is appropriate for this study.

Several studies have demonstrated that hypocalcemia is common in critically ill patients [53] and associated with worse outcomes in conditions such as sepsis, cardiogenic disease, trauma, renal failure, and brain injury [54,55,56]. In cardiac arrest, lower calcium levels may indicate poor perfusion, metabolic acidosis, or intracellular ion shifts [17,57]. Akasaka et al. [18] reported that lower adjusted calcium levels within 48 h were associated with poor neurological outcomes after OHCA. Lee et al. [58] proposed an ion shift index that incorporated calcium and other electrolytes as prognostic markers. However, prior studies have not systematically evaluated the prognostic value of serial ionized calcium measurements while adjusting for calcium administration. Our study addressed this gap by demonstrating the added value of longitudinal calcium monitoring. Although the absolute difference in adjusted ionized calcium levels between outcome groups was numerically small, this difference remained statistically and physiologically relevant. Even mild deviations in ionized calcium within or slightly below the normal physiological range (approximately 4.5–5.3 mg/dL) may indicate disrupted calcium homeostasis in critically ill patients. Previous studies have reported that such subtle decreases in ionized calcium can impair myocardial contractility, vascular tone, and neuronal excitability, potentially influencing post-cardiac arrest recovery [41,42,43]. Therefore, the modest difference observed in our study may still reflect clinically meaningful alterations in calcium regulation after resuscitation.

This study had some limitations. First, it was a single-center retrospective observational study, which may limit the generalizability of our findings. Second, although we adjusted for calcium infusion using both time-specific and cumulative doses, calcium administration was not protocolized and was subject to the physician’s discretion. Therefore, residual confounding due to unmeasured factors cannot be fully excluded. Third, although we used pH-adjusted ionized calcium as the main predictor, ionized calcium values were affected by multiple biochemical parameters, such as lactate, bicarbonate, and phosphate levels, which were not directly corrected in our models. Similarly, other modulators of calcium homeostasis, including parathyroid hormone, 1,25-dihydroxyvitamin D, fibroblast growth factor 23, calcitonin, and calcium-sensing receptor activity, were not measured; thus, their potential impact could not be assessed. Fourth, patients with renal dysfunction, hypertension, or heart failure were included, and medications affecting calcium metabolism were not fully accounted for, which may have introduced additional heterogeneity. Fifth, we reported outcomes at 30 days, which may not capture the long-term neurological prognosis; however, longer follow-up data were not consistently available in our registry. Finally, although we demonstrated an association between calcium levels and outcomes, this study was not designed to determine the causal effects of calcium administration. Prospective interventional studies are needed to clarify whether modulating calcium levels can improve outcomes.

## 5. Conclusions

In this cohort of patients with OHCA treated with TTM, higher adjusted ionized calcium levels during the early post-ROSC period were consistently associated with better neurological outcomes and lower 30-day mortality. These associations remained robust even after adjusting for calcium infusion and other clinical covariates. Although dynamic calcium trends showed limited prognostic value, higher adjusted ionized calcium levels across time points were consistently associated with favorable outcomes, underscoring their potential as reliable prognostic indicators. Our findings support the clinical relevance of adjusted ionized calcium levels in post-cardiac arrest care and highlight the need for further prospective research to clarify their therapeutic implications.

## Figures and Tables

**Table 1:** Clinical and laboratory characteristics of the study population by neurological outcome and 30-day mortality.

	Neurological Outcome at 30 Days			30-Day Mortality		
	Favorable (n = 181, 42.9%)	Unfavorable (n = 240, 57.1%)	*p*-Value	Survived (n = 263, 62.5%)	Dead (n = 158, 37.5%)	*p*-Value
**Patient characteristics**						
Age, years	56.4 ± 16.0	59.2 ± 17.1	0.089	56.5 ± 16.4	60.5 ± 16.9	0.017
Male, *n* (%)	152 (84.0%)	161 (67.1%)	<0.001	206 (78.3%)	107 (67.7%)	0.022
**Cardiac arrest information**						
Witnessed, *n* (%)	137 (75.7%)	148 (61.7%)	0.003	195 (74.1%)	90 (57.0%)	<0.001
Bystander, *n* (%)	140 (77.3%)	149 (62.1%)	0.001	190 (72.2%)	99 (62.7%)	0.052
Non-cardiac cause, *n* (%)	146 (80.7%)	97 (40.4%)	<0.001	177 (67.3%)	66 (41.8%)	<0.001
Initial rhythm, shockable (VF/VT), *n* (%)	114 (63.0%)	52 (21.7%)	<0.001	136 (51.7%)	30 (19.0%)	<0.001
Defibrillation, *n* (%)	72 (40.0%)	47 (19.6%)	<0.001	87 (33.2%)	32 (20.3%)	<0.001
Total dose of epinephrine, mg	1.6 ± 2.8	2.8 ± 3.1	<0.001	1.9 ± 2.8	2.9 ± 3.3	0.001
Time from collapse to ROSC, mins	21.1 ± 17.3	33.5 ± 18.5	<0.001	24.0 ± 17.6	35.2 ± 19.3	<0.001
**Comorbidities, *n* (%)**						
Hypertension	63 (34.8%)	96 (40.0%)	0.324	89 (33.8%)	70 (44.3%)	0.041
Diabetes mellitus	30 (16.6%)	70 (29.2%)	0.004	54 (20.5%)	46 (29.1%)	0.059
Coronary artery disease	52 (28.7%)	45 (18.8%)	0.022	62 (23.6%)	35 (22.2%)	0.829
Pulmonary disease	12 (6.6%)	24 (10.0%)	0.295	24 (9.1%)	12 (7.6%)	0.716
Renal disease	10 (5.5%)	46 (19.2%)	<0.001	20 (7.6%)	36 (22.8%)	<0.001
Cerebrovascular accident	13 (7.2%)	22 (9.2%)	0.581	15 (5.7%)	20 (12.7%)	0.02
Cardiac failure	79 (43.6%)	153 (63.8%)	<0.001	123 (46.8%)	109 (69.0%)	<0.001
**Hospital care**						
CRRT	12 (6.6%)	68 (28.3%)	<0.001	24 (9.1%)	56 (35.4%)	<0.001
ECMO	21 (11.6%)	20 (8.3%)	0.34	28 (10.6%)	13 (8.2%)	0.522
**Adjusted ionized calcium level, mg/dL**						
0 h after ROSC	1.02 ± 0.12	0.92 ± 0.11	<0.001	0.99 ± 0.12	0.91 ± 0.11	<0.001
12 h after ROSC	1.15 ± 0.08	1.06 ± 0.11	<0.001	1.13 ± 0.09	1.06 ± 0.12	<0.001
24 h after ROSC	1.18 ± 0.07	1.11 ± 0.10	<0.001	1.16 ± 0.08	1.11 ± 0.11	<0.001
48 h after ROSC	1.19 ± 0.07	1.14 ± 0.10	<0.001	1.18 ± 0.08	1.14 ± 0.11	0.001
72 h after ROSC	1.21 ± 0.07	1.18 ± 0.10	0.003	1.20 ± 0.07	1.18 ± 0.10	0.086
Calcium infusion, yes	176 (97.2%)	207 (86.2%)	<0.001	247 (93.9%)	136 (86.1%)	0.011
Calcium infusion, total dose, mg	3329.8 ± 2140.8	1812.1 ± 1485.9	<0.001	2846.0 ± 2136.5	1829.8 ± 1364.9	<0.001

Bold indicates section headings within the table. The data are presented as the means ± standard deviations or numbers (%), as appropriate. Abbreviations: VF, ventricular fibrillation; VT, ventricular tachycardia; CRRT, continuous renal replacement therapy; ECMO, extracorporeal membrane oxygenation.

**Table 2:** Comparison of the AIC and AUC values for multivariable models including adjusted ionized calcium levels at each time point, corrected for prior calcium infusion dose.

	Neurological Outcome at 30 Days		30-Day Mortality	
	AIC Goodness-Of-Fit	AUC (95% CI)	AIC Goodness-Of-Fit	AUC (95% CI)
Excluding Ca^2+^ level and calcium infusion variables	427.35	0.84 (0.80–0.88)	469.70	0.78 (0.74–0.83)
Ca^2+^ at 0 h	403.09	0.86 (0.83–0.90)	457.08	0.80 (0.75–0.84)
Ca^2+^ at 12 h (corrected for 0–12 h infusion)	367.19	0.88 (0.84–0.91)	424.79	0.81 (0.77–0.86)
Ca^2+^ at 24 h (corrected for 12–24 h infusion)	379.83	0.87 (0.84–0.91)	445.75	0.80 (0.76–0.84)
Ca^2+^ at 48 h (corrected for 24–48 h infusion)	362.87	0.88 (0.85–0.92)	432.84	0.80 (0.77–0.85)
Ca^2+^ at 72 h (corrected for 48–72 h infusion)	386.58	0.86 (0.83–0.90)	425.76	0.80 (0.75–0.84)

Each model included adjusted ionized calcium levels at a specific time point (0, 12, 24, 48, or 72 h post-ROSC), corrected for calcium infusion during the prior interval. All models were adjusted for other covariates associated with the outcome. Abbreviations: AIC, Akaike information criterion; AUC, area under the receiver operating characteristic curve; CI, confidence interval; ROSC, return of spontaneous circulation.

**Table 3:** Multivariable logistic regression models for neurological outcomes and 30-day mortality, incorporating baseline and slope of adjusted ionized calcium from a linear mixed model.

	Neurological Outcome at 30 Days				30-Day Mortality			
	Univariable		Multivariable		Univariable		Multivariable	
	OR (95% CI)	*p*-Value	OR (95% CI)	*p*-Value	OR (95% CI)	*p*-Value	OR (95% CI)	*p*-Value
Initial rhythm	0.16 (0.11–0.25)	<0.001	0.53 (0.28–1.00)	0.049	0.22 (0.14–0.35)	<0.001	0.43 (0.23–0.82)	0.010
Time-to-ROSC	1.04 (1.03–1.06)	<0.001	1.05 (1.03–1.07)	<0.001	1.03 (1.02–1.05)	<0.001	1.03 (1.01–1.04)	<0.001
Non-cardiac cause	0.16 (0.10–0.25)	<0.001	0.42 (0.21–0.85)	0.016	0.35 (0.23–0.52)	<0.001	1.20 (0.64–2.27)	0.577
History of renal disease	4.05 (2.06–8.74)	<0.001	3.52 (1.41–9.50)	0.009	3.59 (2.01–6.56)	<0.001	3.93 (1.96–8.13)	<0.001
Sex	2.57 (1.61–4.21)	<0.001	2.78 (1.37–5.76)	0.005	1.72 (1.10–2.69)	0.016		
Witnessed	0.52 (0.34–0.79)	0.002	0.75 (0.39–1.41)	0.365	0.46 (0.30–0.70)	<0.001	0.62 (0.37–1.04)	0.070
Baseline Ca^2+^	0.86 (0.83–0.89)	<0.001	0.83 (0.76–0.90)	<0.001	0.90 (0.87–0.93)	<0.001	0.90 (0.84–0.96)	0.001
Rate of change in Ca^2+^	1.49 (1.32–1.69)	<0.001	1.04 (0.78–1.38)	0.805	1.36 (1.22–1.53)	<0.001	1.02 (0.82–1.27)	0.873
Total calcium infusion dose	0.95 (0.94–0.97)	<0.001	0.93 (0.91–0.95)	<0.001	0.97 (0.96–0.98)	<0.001	0.96 (0.95–0.98)	<0.001

Abbreviations: ROSC, return of spontaneous circulation; OR, odds ratio; CI, confidence interval.

**Table 4:** Comparisons of different multivariable logistic regression models for neurological outcomes and 30-day mortality.

Models	Neurological Outcome at 30 Days		30-Day Mortality		Comparison
	AUC (95% CI)	*p*-Value	AUC (95% CI)	*p*-Value	
1. Excluding calcium-related variables	0.84 (0.80–0.88)	<0.001	0.78 (0.74–0.83)	0.012	Model 1 vs. 2
2. Neurological outcome: Ca^2+^ at 48 h (corrected for 24–48 h infusion) Mortality outcome: Ca^2+^ at 12 h (corrected for 0–12 h infusion)	0.88 (0.85–0.92)	0.005	0.82 (0.77–0.86)	0.124	Model 2 vs. 3
3. Baseline Ca^2+^ and slope over time (corrected for cumulative infusion)	0.92 (0.89–0.94)	<0.001	0.83 (0.79–0.87)	0.001	Model 1 vs. 3

Abbreviations: AUC, area under the receiver operating characteristic curve; CI, confidence interval.

## Data Availability

The datasets presented in this article are not readily available because of institutional privacy restrictions. Requests to access the datasets should be directed to the corresponding author on reasonable request and with permission from the Institutional Review Board of Yonsei University College of Medicine, Severance Hospital.

## Supplementary Materials

The following supporting information can be downloaded at https://www.mdpi.com/article/10.3390/life15121889/s1, Tables S1 and S2. Multivariable logistic regression models assessing the association between adjusted ionized calcium levels (measured at each time point post-ROSC) and neurological outcome or 30-day mortality, correcting for the preceding period’s calcium infusion dose and other significant clinical covariates. Table S3. Distribution of serum electrolytes and related biochemical parameters according to neurological outcomes and 30-day mortality. Table S4. Multivariable logistic regression models of neurological (Cerebral Performance Category) and mortality outcomes incorporating adjusted Ca^2+^ levels (0, 12, 24, 48, and 72 h) as estimated baseline Ca^2+^ and slope of Ca^2+^ over time in a linear mixed model, while adjusting for total calcium infusion dose and other predictor variables associated with the outcome (including other serum electrolytes and proteins).

## Abbreviations

The following abbreviations are used in this manuscript:

OHCA	Out-of-hospital cardiac arrest
TTM	Targeted temperature management
ROSC	Return of spontaneous circulation
CPC	Cerebral Performance Category
OR	Odds ratio
CI	Confidence interval
AUC	Area under the receiver operating characteristic curve
CP	Critical pathway
CPR	Cardiopulmonary resuscitation
