# Supplementary material for: Association Between Serum Ionized Calcium Levels and Neurological Outcomes in Patients with Out-of-Hospital Cardiac Arrest
Source: Life (Basel). 2025 Dec 10;15(12):1889. doi: 10.3390/life15121889 (PMC12734376; doi:10.3390/life15121889)
Supplement: Supplementary file 1 [file life-15-01889-s001.zip › life-3932215-Supplementary Tables.pdf]

Supplementary Tables

Tables S1 and S2. Multivariable logistic regression models assessing the association between adjusted ionized calcium levels (measured at each time point post-ROSC) and neurological outcome or 30-day mortality, correcting for the preceding period's calcium infusion dose and other significant clinical covariates

Table S1-1. Multivariable logistic regression model of neurological outcome at 30 days, including other predictor variables associated with the outcome, but without any calcium-related variables included

	**OR**	**Lower 95% CI**	**Upper 95% CI**	**P-value**
Initial rhythm	0.40	0.23	0.69	<0.001
Time-to-ROSC	1.05	1.03	1.06	<0.001
Non-cardiac cause	0.24	0.13	0.42	<0.001
History of renal disease	3.18	1.45	7.54	0.006
Sex	1.97	1.11	3.57	0.022
Witnessed	0.58	0.34	0.99	0.045

Abbreviations: ROSC, return of spontaneous circulation; OR, odds ratio; CI, confidence interval

Table S2-1. Multivariable logistic regression model of mortality at 30 days, including other predictor variables associated with the outcome, but without any calcium-related variables included

	**OR**	**Lower 95% CI**	**Upper 95% CI**	**P-value**
Initial rhythm	0.37	0.21	0.66	0.001
Time-to-ROSC	1.03	1.02	1.05	<0.001
Non-cardiac cause	0.59	0.34	0.99	0.046
History of renal disease	3.23	1.70	6.27	<0.001
Witnessed	0.52	0.32	0.84	0.007

Abbreviations: ROSC, return of spontaneous circulation; OR, odds ratio; CI, confidence interval

Table S1-2. Multivariable logistic regression model assessing 30-day neurological outcome based on baseline (0 h) adjusted ionized calcium level, corrected for other outcome-related covariates

	**OR**	**Lower 95% CI**	**Upper 95% CI**	**P-value**
Initial rhythm	0.48	0.27	0.86	0.013
Time-to-ROSC	1.03	1.02	1.05	<0.001
Non-cardiac cause	0.27	0.14	0.48	<0.001
History of renal disease	4.16	1.81	10.40	0.001
Sex	2.04	1.11	3.79	0.022
Witnessed	0.50	0.29	0.88	0.017
Ca^{2+} at 0 h	0.94	0.92	0.97	<0.001

Abbreviations: ROSC, return of spontaneous circulation; OR, odds ratio; CI, confidence interval

Table S2-2. Multivariable logistic regression model assessing 30-day mortality based on baseline (0 h) adjusted ionized calcium level, corrected for other outcome-related covariates

	**OR**	**Lower 95% CI**	**Upper 95% CI**	**P-value**
Initial rhythm	0.42	0.23	0.76	0.004
Time-to-ROSC	1.03	1.01	1.04	<0.001
Non-cardiac cause	0.70	0.40	1.21	0.195
History of renal disease	3.84	1.98	7.70	<0.001
Witnessed	0.50	0.30	0.81	0.005
Ca^{2+} at 0 h	0.96	0.94	0.99	0.001

Abbreviations: ROSC, return of spontaneous circulation; OR, odds ratio; CI, confidence interval

Table S1-3. Multivariable logistic regression model assessing 30-day neurological outcome based on adjusted ionized calcium level at 12 h, corrected for prior calcium infusion and other outcome-related covariates

	**OR**	**Lower 95% CI**	**Upper 95% CI**	**P-value**
Initial rhythm	0.43	0.24	0.78	0.005
Time-to-ROSC	1.04	1.02	1.06	<0.001
Non-cardiac cause	0.31	0.17	0.58	<0.001
History of renal disease	5.71	2.40	14.90	<0.001
Sex	2.08	1.10	3.99	0.025
Witnessed	0.57	0.31	1.04	0.070

Ca^{2+} at 12 h	0.92	0.89	0.95	<0.001
Calcium infusion (0–12 h)	0.97	0.93	1.00	0.054

Abbreviations: ROSC, return of spontaneous circulation; OR, odds ratio; CI, confidence interval

Table S2-3. Multivariable logistic regression model assessing 30-day mortality based on adjusted ionized calcium level at 12 h, corrected for prior calcium infusion and other outcome-related covariates

	OR	Lower 95% CI	Upper 95% CI	P-value
Initial rhythm	0.41	0.22	0.75	0.004
Time-to-ROSC	1.03	1.01	1.04	<0.001
Non-cardiac cause	0.73	0.41	1.31	0.289
History of renal disease	4.42	2.21	9.13	<0.001
Witnessed	0.50	0.30	0.84	0.009
Ca^{2+} at 12 h	0.95	0.93	0.97	<0.001
Calcium infusion (0–12 h)	0.98	0.95	1.02	0.327

Abbreviations: ROSC, return of spontaneous circulation; OR, odds ratio; CI, confidence interval

Table S1-4. Multivariable logistic regression model assessing 30-day neurological outcome based on adjusted ionized calcium level at 24 h, corrected for prior calcium infusion and other outcome-related covariates

	OR	Lower 95% CI	Upper 95% CI	P-value
Initial rhythm	0.40	0.22	0.72	0.002
Time-to-ROSC	1.05	1.03	1.06	<0.001
Non-cardiac cause	0.32	0.17	0.60	<0.001
History of renal disease	4.07	1.76	10.20	0.002
Sex	2.31	1.23	4.47	0.011
Witnessed	0.64	0.35	1.14	0.131
Ca^{2+} at 24 h	0.93	0.90	0.96	<0.001
Calcium infusion (12–24 h)	0.95	0.91	0.98	0.004

Abbreviations: ROSC, return of spontaneous circulation; OR, odds ratio; CI, confidence interval

Table S2-4. Multivariable logistic regression model assessing 30-day mortality based on adjusted ionized calcium level at 24 h, corrected for prior calcium infusion and other outcome-related covariates

	**OR**	**Lower 95% CI**	**Upper 95% CI**	**P-value**
Initial rhythm	0.38	0.21	0.69	0.002
Time-to-ROSC	1.03	1.02	1.04	<0.001
Non-cardiac cause	0.71	0.40	1.26	0.237
History of renal disease	4.52	2.30	9.14	<0.001
Witnessed	0.60	0.36	0.99	0.044
Ca^{2+} at 24 h	0.96	0.93	0.98	0.001
Calcium infusion (12–24 h)	1.00	0.96	1.03	0.867

Abbreviations: ROSC, return of spontaneous circulation; OR, odds ratio; CI, confidence interval

Table S1-5. Multivariable logistic regression model assessing 30-day neurological outcome based on adjusted ionized calcium level at 48 h, corrected for prior calcium infusion and other outcome-related covariates

	**OR**	**Lower 95% CI**	**Upper 95% CI**	**P-value**
Initial rhythm	0.33	0.18	0.60	<0.001
Time-to-ROSC	1.07	1.05	1.09	<0.001
Non-cardiac cause	0.34	0.18	0.65	0.001
History of renal disease	2.97	1.27	7.51	0.015
Sex	2.78	1.45	5.50	0.003
Witnessed	0.63	0.34	1.14	0.129
Ca^{2+} at 48 h	0.92	0.89	0.96	<0.001
Calcium infusion (24–48 h)	0.90	0.86	0.94	<0.001

Abbreviations: ROSC, return of spontaneous circulation; OR, odds ratio; CI, confidence interval

Table S2-5. Multivariable logistic regression model assessing 30-day mortality based on adjusted ionized calcium level at 48 h, corrected for prior calcium infusion and other outcome-related covariates

	**OR**	**Lower 95% CI**	**Upper 95% CI**	**P-value**
Initial rhythm	0.37	0.20	0.69	0.002
Time-to-ROSC	1.04	1.03	1.06	<0.001
Non-cardiac cause	0.86	0.47	1.56	0.613
History of renal disease	3.55	1.81	7.14	<0.001

Witnessed	0.58	0.35	0.96	0.035
Ca^{2+} at 48 h	0.96	0.93	0.98	0.001
Calcium infusion (24–48 h)	0.95	0.91	0.98	0.003

Abbreviations: ROSC, return of spontaneous circulation; OR, odds ratio; CI, confidence interval

Table S1-6. Multivariable logistic regression model assessing 30-day neurological outcome based on adjusted ionized calcium level at 72 h, corrected for prior calcium infusion and other outcome-related covariates

	**OR**	**Lower 95% CI**	**Upper 95% CI**	**P-value**
Initial rhythm	0.38	0.21	0.68	0.001
Time-to-ROSC	1.05	1.03	1.07	<0.001
Non-cardiac cause	0.27	0.15	0.50	<0.001
History of renal disease	2.42	1.07	5.86	0.039
Sex	2.10	1.14	3.93	0.018
Witnessed	0.69	0.39	1.22	0.200
Ca^{2+} at 72 h	0.97	0.94	1.00	0.034
Calcium infusion (48–72 h)	0.90	0.86	0.94	<0.001

Abbreviations: ROSC, return of spontaneous circulation; OR, odds ratio; CI, confidence interval

Table S2-6. Multivariable logistic regression model assessing 30-day mortality based on adjusted ionized calcium level at 72 h, corrected for prior calcium infusion and other outcome-related covariates

	**OR**	**Lower 95% CI**	**Upper 95% CI**	**P-value**
Initial rhythm	0.35	0.18	0.65	0.001
Time-to-ROSC	1.03	1.02	1.05	<0.001
Non-cardiac cause	0.81	0.45	1.45	0.469
History of renal disease	2.96	1.52	5.87	0.002
Witnessed	0.60	0.36	0.99	0.047
Ca^{2+} at 72 h	0.98	0.95	1.01	0.157
Calcium infusion (48–72 h)	0.92	0.87	0.96	0.001

Abbreviations: ROSC, return of spontaneous circulation; OR, odds ratio; CI, confidence interval

Tables S3. Distribution of serum electrolytes and related biochemical parameters according to neurological outcomes and 30-day mortality

	Neurological outcome at 30-days			30-day mortality		
	Favorable (n=181, 42.9%)	Unfavorable (n=240, 57.1%)	P-value	Survival (n=263, 62.5%)	death (n=158, 37.5%)	P-value
**Serum sodium level**						
0 h after ROSC	139.0 ± 10.3	138.7 ± 10.5	0.777	139.2 ± 9.2	138.3 ± 12.2	0.421
12 h after ROSC	141.7 ± 3.8	141.9 ± 6.2	0.586	141.6 ± 4.5	142.2 ± 6.5	0.297
24 h after ROSC	141.3 ± 3.8	142.6 ± 6.4	0.010	141.5 ± 4.5	142.9 ± 6.8	0.021
48 h after ROSC	140.0 ± 4.0	141.1 ± 11.2	0.163	139.9 ± 9.6	142.0 ± 7.0	0.011
72 h after ROSC	139.2 ± 10.4	140.3 ± 15.1	0.402	139.2 ± 12.7	140.9 ± 14.1	0.223
**Serum potassium level**						
0 h after ROSC	4.9 ± 9.9	4.8 ± 1.2	0.818	4.8 ± 8.2	4.8 ± 1.3	0.981
12 h after ROSC	5.4 ± 10.9	4.0 ± 0.8	0.084	5.0 ± 9.0	4.0 ± 0.8	0.099
24 h after ROSC	4.3 ± 2.7	4.0 ± 0.7	0.146	4.2 ± 2.3	4.1 ± 0.7	0.675
48 h after ROSC	4.1 ± 0.6	5.0 ± 7.8	0.078	4.6 ± 7.0	4.5 ± 3.3	0.934
72 h after ROSC	3.9 ± 0.5	4.4 ± 6.4	0.288	3.9 ± 0.5	4.7 ± 8.0	0.226
**Serum chloride level**						
0 h after ROSC	101.0 ± 11.0	100.1 ± 8.9	0.380	100.9 ± 9.7	100.0 ± 10.0	0.372
12 h after ROSC	105.1 ± 8.6	105.2 ± 6.8	0.878	105.2 ± 7.9	105.1 ± 7.2	0.931
24 h after ROSC	105.8 ± 4.2	105.9 ± 6.8	0.888	106.0 ± 4.9	105.6 ± 7.2	0.560
48 h after ROSC	103.2 ± 8.4	103.1 ± 14.3	0.930	103.1 ± 11.3	103.3 ± 13.3	0.821
72 h after ROSC	103.8 ± 4.7	104.2 ± 12.4	0.613	104.1 ± 5.4	104.0 ± 14.8	0.962

**Serum magnesium level**						
0 h after ROSC	1.6 ± 0.6	1.8 ± 0.7	0.006	1.7 ± 0.5	1.8 ± 0.8	0.098
12 h after ROSC	1.5 ± 0.3	1.4 ± 0.3	0.039	1.5 ± 0.3	1.4 ± 0.3	0.149
24 h after ROSC	1.5 ± 0.2	1.9 ± 8.5	0.380	1.4 ± 0.2	2.3 ± 10.6	0.350
48 h after ROSC	1.5 ± 0.5	1.9 ± 7.4	0.366	1.9 ± 6.9	1.5 ± 0.7	0.384
72 h after ROSC	1.5 ± 0.2	1.5 ± 0.3	0.241	1.5 ± 0.3	1.5 ± 0.3	0.474
**Serum phosphate level**						
0 h after ROSC	6.1 ± 2.1	7.9 ± 2.3	<0.001	6.6 ± 2.3	7.9 ± 2.4	<0.001
12 h after ROSC	3.7 ± 1.3	4.1 ± 2.0	0.083	3.7 ± 1.5	4.1 ± 2.2	0.148
24 h after ROSC	3.3 ± 1.1	3.7 ± 1.8	0.016	3.4 ± 1.2	3.8 ± 2.0	0.033
48 h after ROSC	3.3 ± 1.0	3.7 ± 1.7	0.002	3.3 ± 1.1	3.8 ± 1.8	0.004
72 h after ROSC	2.9 ± 1.0	3.2 ± 1.7	0.034	2.9 ± 1.1	3.3 ± 1.9	0.043
**Serum albumin level**						
0 h after ROSC	3.7 ± 0.6	3.4 ± 0.6	<0.001	3.6 ± 0.6	3.3 ± 0.6	<0.001
12 h after ROSC	3.3 ± 0.6	3.0 ± 0.6	<0.001	3.3 ± 0.6	2.9 ± 0.6	<0.001
24 h after ROSC	3.2 ± 0.5	2.9 ± 0.5	<0.001	3.1 ± 0.5	2.9 ± 0.5	0.001
48 h after ROSC	3.2 ± 0.4	2.9 ± 0.4	<0.001	3.1 ± 0.4	2.8 ± 0.5	<0.001
72 h after ROSC	3.1 ± 0.4	2.8 ± 0.4	<0.001	3.0 ± 0.4	2.8 ± 0.4	<0.001
**Serum lactate level**						
0 h after ROSC	9.6 ± 4.7	11.6 ± 4.9	<0.001	10.1 ± 4.8	11.8 ± 5.0	0.001
12 h after ROSC	2.8 ± 2.1	4.3 ± 3.3	<0.001	2.9 ± 2.2	4.7 ± 3.6	<0.001
24 h after ROSC	1.9 ± 1.2	3.8 ± 3.5	<0.001	2.2 ± 2.0	4.2 ± 3.6	<0.001
48 h after ROSC	1.5 ± 1.3	2.6 ± 2.8	0.001	1.6 ± 1.2	3.1 ± 3.2	<0.001
72 h after ROSC	1.2 ± 0.7	2.0 ± 2.8	0.009	1.3 ± 0.8	2.3 ± 3.3	0.020

Abbreviations: ROSC, return of spontaneous circulation

Tables S4. Multivariable logistic regression models of neurological (Cerebral Performance Category) and mortality outcomes incorporating adjusted Ca^{2+} levels (0, 12, 24, 48, and 72 h) as estimated baseline Ca^{2+} and slope of Ca^{2+} over time in a linear mixed model, while adjusting for total calcium infusion dose and other predictor variables associated with the outcome (including other serum electrolytes and proteins)

	Neurological outcome at 30-days				30-day mortality			
Variables	Univariable		Multivariable		Univariable		Multivariable	
	OR (95% CI)	P-value	OR (95% CI)	P-value	OR (95% CI)	P-value	OR (95% CI)	P-value
Initial rhythm	0.16 (0.11–0.25)	<0.001	0.51 (0.25–1.07)	0.074	0.22 (0.14–0.35)	<0.001	0.43 (0.21–0.85)	0.016
Time-to-ROSC	1.04 (1.03–1.06)	<0.001	1.04 (1.02–1.06)	<0.001	1.03 (1.02–1.05)	<0.001	1.02 (1.01–1.04)	0.006
Non-cardiac cause	0.16 (0.10–0.25)	<0.001	0.65 (0.29–1.49)	0.307	0.35 (0.23–0.52)	<0.001	1.39 (0.71–2.78)	0.349
Renal event	4.05 (2.06–8.74)	<0.001	3.93 (1.30–13.5)	0.020	3.59 (2.01–6.56)	<0.001	3.98 (1.87–8.84)	<0.001
Gender	2.57 (1.61–4.21)	<0.001	2.63 (1.19–6.00)	0.018	1.72 (1.10–2.69)	0.016	-	-
Witnessed	0.52 (0.34–0.79)	0.002	1.00 (0.48–2.08)	0.997	0.46 (0.30–0.70)	<0.001	0.64 (0.37–1.11)	0.115
Baseline sodium	1.00 (0.98–1.02)	0.778	1.00 (0.93–1.03)	0.863	0.99 (0.97–1.01)	0.403	0.99 (0.95–1.02)	0.435
Baseline potassium	1.00 (0.96–1.03)	0.794	0.99 (0.89–1.06)	0.901	1.00 (0.96–1.04)	0.985	0.99 (0.87–1.04)	0.735
Baseline chloride	0.99 (0.97–1.01)	0.374	0.98 (0.95–1.01)	0.254	0.99 (0.97–1.01)	0.380	0.99 (0.97–1.02)	0.581
Baseline magnesium	1.97 (1.24–3.32)	0.008	2.47 (1.04–6.32)	0.049	1.34 (0.98–1.96)	0.091	1.25 (0.73–2.23)	0.424
Baseline phosphate	1.47 (1.32–1.64)	<0.001	1.47 (1.20–1.83)	<0.001	1.26 (1.15–1.38)	<0.001	1.07 (0.93–1.23)	0.355
Baseline albumin	0.29 (0.19–0.43)	<0.001	0.60 (0.32–1.14)	0.124	0.40 (0.28–0.57)	<0.001	0.69 (0.42–1.10)	0.118
Baseline lactate	1.09 (1.05–1.14)	<0.001	0.87 (0.79–0.96)	0.005	1.07 (1.03–1.12)	0.001	0.99 (0.93–1.06)	0.721
Baseline Ca^{2+}	0.86 (0.83–0.89)	<0.001	0.80 (0.72–0.88)	<0.001	0.90 (0.87–0.93)	<0.001	0.90 (0.84–0.96)	0.003
Rate of Ca^{2+} change over time	1.49 (1.32–1.69)	<0.001	0.95 (0.66–1.35)	0.789	1.36 (1.22–1.53)	<0.001	0.96 (0.75–1.23)	0.769
Total calcium infusion dose	0.95 (0.94–0.97)	<0.001	0.92 (0.89–0.94)	<0.001	0.97 (0.96–0.98)	<0.001	0.96 (0.95–0.98)	<0.001

Abbreviations: ROSC, return of spontaneous circulation; OR, odds ratio; CI, confidence interval
